# Method to Reduce Long-lived Fission Products by Nuclear Transmutations with Fast Spectrum Reactors

**DOI:** 10.1038/s41598-017-14319-7

**Published:** 2017-10-24

**Authors:** Satoshi Chiba, Toshio Wakabayashi, Yoshiaki Tachi, Naoyuki Takaki, Atsunori Terashima, Shin Okumura, Tadashi Yoshida

**Affiliations:** 1 Laboratory for Advanced Nuclear Energy, Tokyo Institute of Technology, 2–12–1 Ookayama, Meguro-ku, Tokyo, 152–8550 Japan; 2 Tohoku University, 2–1–1 Katahira, Aoba-ku, Sendai, Miyagi, 980–8577 Miyagi, Japan; 3 Oarai Research and Development Center, Japan Atomic Energy Agency, 4002, Narita-cho, Oaraimachi, Ibaraki, 311–1393 Japan; 4 Department of Nuclear Safety Engineering, Tokyo City University, 1–28–1 Tamazutsumi, Setagaya-ku, Tokyo, 158–8557 Japan

## Abstract

Transmutation of long-lived fission products (LLFPs: ^79^Se, ^93^Zr, ^99^Tc, ^107^Pd, ^129^I, and ^135^Cs) into short-lived or non-radioactive nuclides by fast neutron spectrum reactors without isotope separation has been proposed as a solution to the problem of radioactive wastes disposal. Despite investigation of many methods, such transmutation remains technologically difficult. To establish an effective and efficient transmutation system, we propose a novel neutron moderator material, yttrium deuteride (YD_2_), to soften the neutron spectrum leaking from the reactor core. Neutron energy spectra and effective half-lives of LLFPs, transmutation rates, and support ratios were evaluated with the continuous-energy Monte Carlo code MVP-II/MVP-BURN and the JENDL–4.0 cross section library. With the YD_2_ moderator in the radial blanket and shield regions, effective half-lives drastically decreased from 10^6^ to 10^2^ years and the support ratios reached 1.0 for all six LLFPs. This successful development and implementation of a transmutation system for LLFPs without isotope separation contributes to a the ability of fast spectrum reactors to reduce radioactive waste by consuming their own LLFPs.

## Introduction

Nuclear energy provides 16% of the world’s electricity production^1^. Although nuclear energy does not release greenhouse gases, it does produce highly radioactive spent nuclear fuel (SNF)^2^. After recovering U and Pu from SNF by conventional reprocessing, such as the PUREX process, the disposal of high-level radioactive wastes is a major concern in many countries. Most of the radioactive hazard remaining in high-level radioactive wastes after thousands of years comes from minor actinides (MAs; isotopes of Np, Am, and Cm) and some long-lived fission products (LLFPs; ^79^Se, ^93^Zr, ^99^Tc, ^107^Pd, ^129^I, and ^135^Cs)^3^. The disposal of such SNF in stable deep geological repository has been considered, however, it has not been fully implemented.

The partitioning and transmutation (P&T) strategy has been proposed and research and development have begun in several countries to reduce the inventory of radioactive wastes to be stored and the long-term hazard for future generations^4–14^. The P&T strategy consists of two major techniques, elemental separation of MAs and LLFPs from high-level radioactive wastes by a chemical process (partitioning) and nuclear reactions of these nuclides into short-lived or non-radioactive nuclides (trasmutation) in nuclear reactors or accelerator-driven transmutation systems(ADS). The P&T strategy has been well studied for MAs^12,15–20^, while little attention has been paid to LLFPs especially for transmutation of LLFPs. After the amount of MAs is reduced by transmutation, LLFPs become the main source of the hazard. It is because they are associated with radioactive release from geological repositories owing to their high solubility in water and mobility in the geosphere^15,21,22^.

There are two technical difficulties that make transmutation of LLFPs less efficient: (1) the isotopic compositions and (2) small neutron capture cross sections of LLFPs^23–25^. The transmutation of LLFPs is a neutron-consuming process and it requires high neutron flux and excess of neutrons in principle. After partitioning, the isotopic compositions of the LLFPs are not monoisotopic, but are a mixture of stable and radioactive isotopes including LLFP. The consumption of neutrons to transmute the LLFPs is much larger than when isotope separation is applied because isotopes other than the LLFPs also capture neutrons. Only ^129^I and ^99^Tc have been considered that can be transmuted because of their relatively large neutron capture cross sections and large isotopic abundance^26,27^. Therefore, in most studies, the transmutation of LLFPs involves isotope separation^14,28–30^. However, no isotope separation system for elements related to LLFPs is technologically and economically feasible at an industrial scale^31,32^.

There are some studies on transmutations of LLFPs by reactor systems to address these two technical difficulties. The feasibility of reactor systems depends on the neutron balance. All LLFPs and the fuel are neutron consumers. Even with isotopic separation, it needs at least 0.3 neutron/fission for LLFP-transmutation^33^. The transmutations of LLFPs in common light water reactors has been known to be difficult mainly because of lack of excess neutrons^34,35^. The subcritical ADS or also called hybrid systems, which produce energy and to transmute radioactive wastes, appear to be a possible alternative to critical reactors^14,16,28,30,36^. Although numerous programs have conducted to evaluate the ADS availability, the high-power accelerator technology is a complex system and any ADS system had not yet been demonstrated.

In this context, fast neutron spectrum reactor is the most attractive solution in the existing system with respect to the neutron balance, because of their high neutron flux and excess of neutrons^29,37–39^. Since most LLFPs have large neutron capture cross sections in the 1 to 10^3^ eV region, the neutron spectrum must be softened to use the fast reactor system for transmuting LLFPs via resonance absorption. Metal hydrides (e.g., ZrH_2_) have been frequently proposed as neutron moderator materials for fast reactor systems because of the good neutron moderating ability of ^1^H and the high density of the materials^40,41^. However, a fission of fissile nuclide (e.g. ^235^U ^239^Pu) and the neutron capture reaction of stable ^92^Zr both produce ^93^Zr, which is one of the LLFPs. Moreover, metal hydrides sometimes induce a thermal spike by elastic collisions due to over-moderated neutrons near the core, causing the dissociation of hydrogen at high temperatures. Therefore, alternative moderator materials are required to develop a more effective transmutation system for LLFPs^41^.

In this study, we propose yttrium deuteride (YD_2_) as a moderator material for the transmutation of LLFPs in a fast spectrum reactor. Yttrium is a possible alternative to Zr because Y has a small neutron capture cross section over a wide neutron energy range and produces no radioactive isotopes with long half-lives. The bulk density of YH_2_ is smaller than that of ZrH_2_ and the dissociation temperature of YH_2_ is 1200 °C, which is 300 °C higher than that of ZrH_2_. These physical and chemical properties may help to avoid thermal spikes^42,43^. Furthermore, we have revealed that the thermal spike observed for ZrH_2_ can be suppressed by using the deuteride (ZrD_2_) instead of the hydride^44^. Based on these findings and the fact that YD_2_ is expected to have similar physical and chemical properties as YH_2_, YD_2_ is a promising material as a neutron moderator for fast spectrum reactors to transmute LLFPs.

To accomplish effective transmutation of LLFPs in reactor systems, the effective half-life, which is the half-life of a nuclide resulting from the transmutation, must be decreased. In addition, the transmutation rate (TR) of LLFP must be larger than the production rate of LLFP in the core. The ratio of the TR to the production rate is called the support ratio (SR), and it is an important parameter to evaluate the efficiency of transmutation systems^26,41^. A transmutation system with SR > 1.0 in any reactor system will prevent the increase of LLFPs from its waste. However, it has not been achieved to transmute all LLFPs with SR > 1.0 without isotope separation.

This paper demonstrates a transmutation method for LLFPs that does not require isotope separations. In this method, the LLFPs can be transmuted to stable or short-lived radioactive isotopes by introducing a novel moderator material, YD_2_, to the fast neutron spectrum reactor. We studied the effects of the moderator on the associated neutron energy spectra, the effective half-lives of LLFPs, transmutation rates (TRs), and SRs by simulations with the continuous-energy Monte Carlo code MVP-II and the burn-up simulation code MVP-BURN with JENDL-4.0 nuclear data library^45,46^.

## Results and Discussion

### Implementation of the concept at a fast reactor

**Figure 1:**
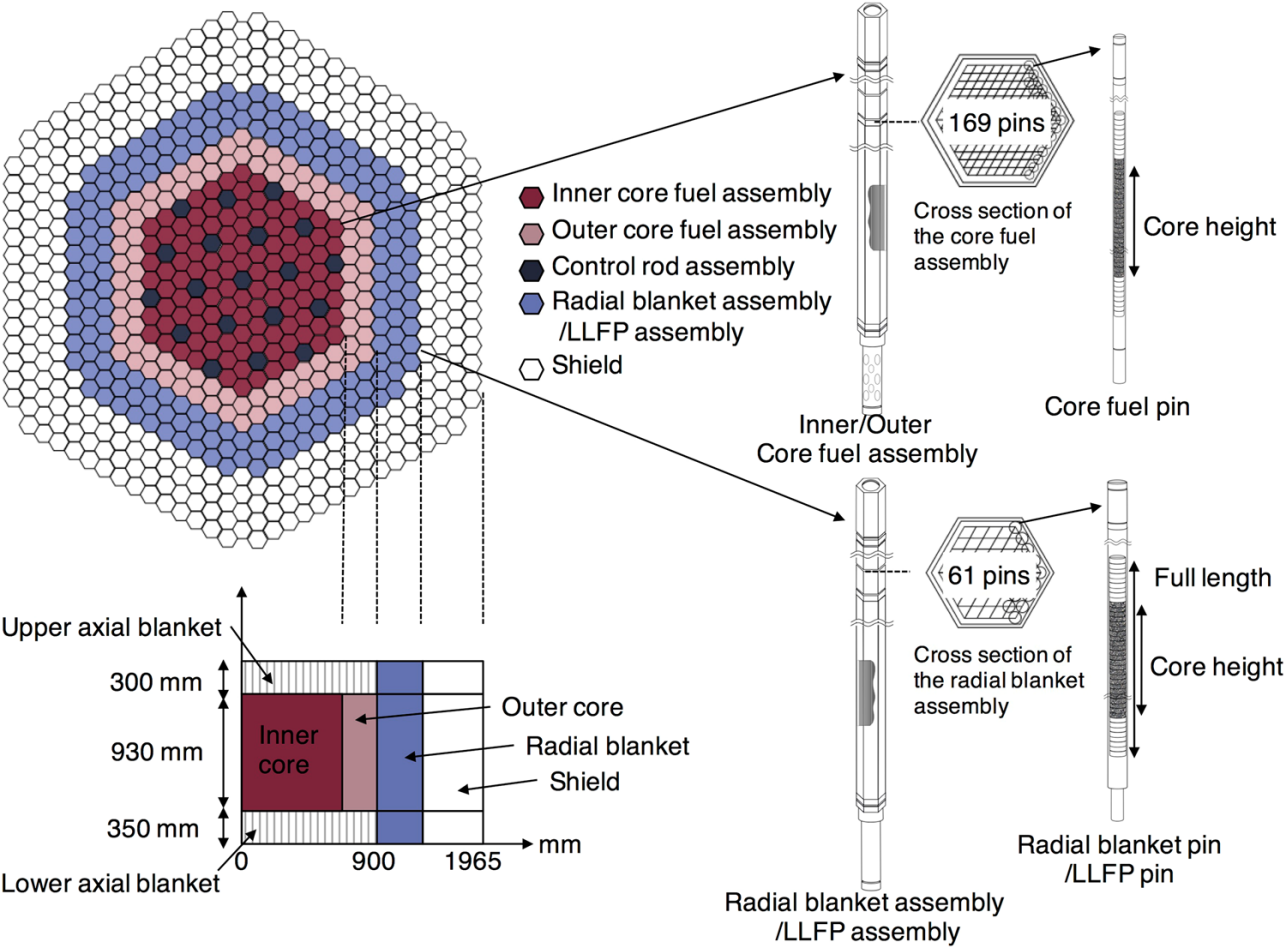
Core layout of the fast spectrum reactor for LLFP transmutation. Plan view and side view of the core layout of the fast spectrum reactor with numbers and lengths of assemblies and pins in the core and radial blanket region. For the transmutations of Cs and Zr, the pellets were loaded along the full length of the radial blanket assembly, whereas the pellets were loaded at the core height of the radial blanket assembly for Se, Tc, Pd, and I. The shield region was used for the transmutations of Cs and Zr, but they were loaded only at the core height of the shield assembly. Unless the shield region is used for the transmutation, the shielding assemblies surround the core and radial blanket assemblies to shield neutrons and gamma rays from the reactor vessel.

Figure 1 shows layout of the fast reactor core, the assembly configuration, and the pin array based on the Japanese fast breeder reactor MONJU (see Supplementary Table 1 for details of the core description). The radial blanket and shield regions where fast neutrons leak from the core are used for transmuting LLFPs. The LLFP assembly is an assembly that are loaded in the radial blanket or shield regions with pins containing pellets of a mixture of LLFPs and moderator. The composition of the SNF depends on the fuel, core type, and irradiation history. The initial isotopic compositions (%) of the elements, including LLFPs, are determined from the burn-up simulation of mixed oxide (MOX) fuel in the fast reactor by MVP-II and MVP-BURN^45,46^. The initial isotopic compositions used in this study are listed in Table 1. In the simulations, the LLFP pellets are assumed as homogeneous mixtures of the metallic or chemical forms of the target LLFP and YD_2_ in various ratios (see Method section for details).

**Table 1:** Initial and final isotope compositions of LLFPs in the system.

Element	Isotope	Initial composition (%)	Final composition (%)
Se	^76^Se	0.027	0.016
	^77^Se	2.786	1.12
	^78^Se	5.587	6.49
	^79^Se	13.32	4.75
	^80^Se	22.75	31.00
	^82^Se	55.52	54.89
Tc	^99^Tc	100.00	72.93
Pd	^104^Pd	2.93	2.62
	^105^Pd	35.14	21.64
	^106^Pd	17.86	30.36
	^107^Pd	21.72	14.92
	^108^Pd	17.12	25.49
	^110^Pd	5.24	4.97
Zr	^90^Zr	0.27	0.41
	^91^Zr	11.72	10.80
	^92^Zr	16.11	16.76
	^92^Zr	19.95	17.84
	^94^Zr	22.33	24.53
	^95^Zr	3.04	0.0026
	^96^Zr	26.59	26.06
I	^127^I	23.91	6.03
	^129^I	76.09	42.82
Cs	^133^Cs	31.40	14.9
	^134^Cs	1.52	0.10
	^135^Cs	35.21	7.22
	^136^Cs	0.05	2.9 × 10^−14^
	^137^Cs	31.82	3.45

Initial compositions based on the burn-up simulations of MOX fuel by a fast breeder reactor core at 80 GWd/t for 2 years and final compositions after 20 years of irradiation in this study by MVP-BURN.

### Neutron spectra at different positions in the reactor

**Figure 2:**
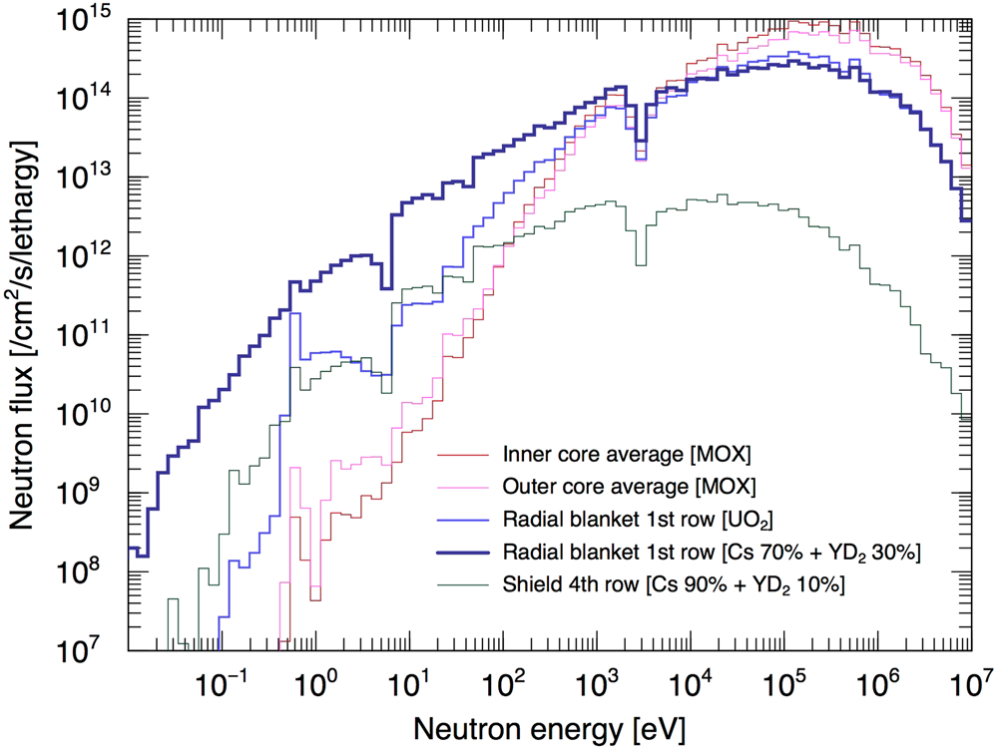
Comparison of neutron spectra at various locations in the reactor. Simulated energy dependence of the neutron flux at the inner- (red), outer- (magenta), first row at the radial blanket with the depleted UO_2_ pellet (blue), first row at the radial blanket with Cs 70% + YD_2_ 30% (bold blue), and outermost row of the shield region with Cs 90% + YD_2_ 10%.

The effect of the proposed YD_2_ moderator material on the neutron spectra leaking from the core was examined. Figure 2 shows the 110-group (110 energy bins) neutron energy spectrum of inner- and outer-cores, the first row in the radial blanket region, and the fourth row in the shield regions. The neutron spectra at the inner- and outer-cores are hard. The neutron spectra at the radial blanket and shield regions broaden and are soften resulting from the introduction of YD_2_ moderator material. The spectrum from the first row of the radial blanket region loaded with depleted UO_2_ pellets (without LLFP and moderator) shows a flux (n cm^−2^s^−1^lethargy^−1^) on the order of 10^12^ at 10–100 eV, whereas the spectrum with LLFP and moderator (Cs 70% + YD_2_ 30%) shows a flux approximately one order of magnitude higher in the energy region below 100 eV. This softened spectrum is effective for transmuting LLFPs. It is also confirmed that the integrals of the microscopic capture reaction rates compared with those without the YD^2^ moderator are greater for all LLFPs (Supplementary Table 3).

### Transmutation of ^79^Se, ^93^Zr, ^99^Tc, ^107^Pd, ^129^I, and ^135^Cs

**Figure 3:**
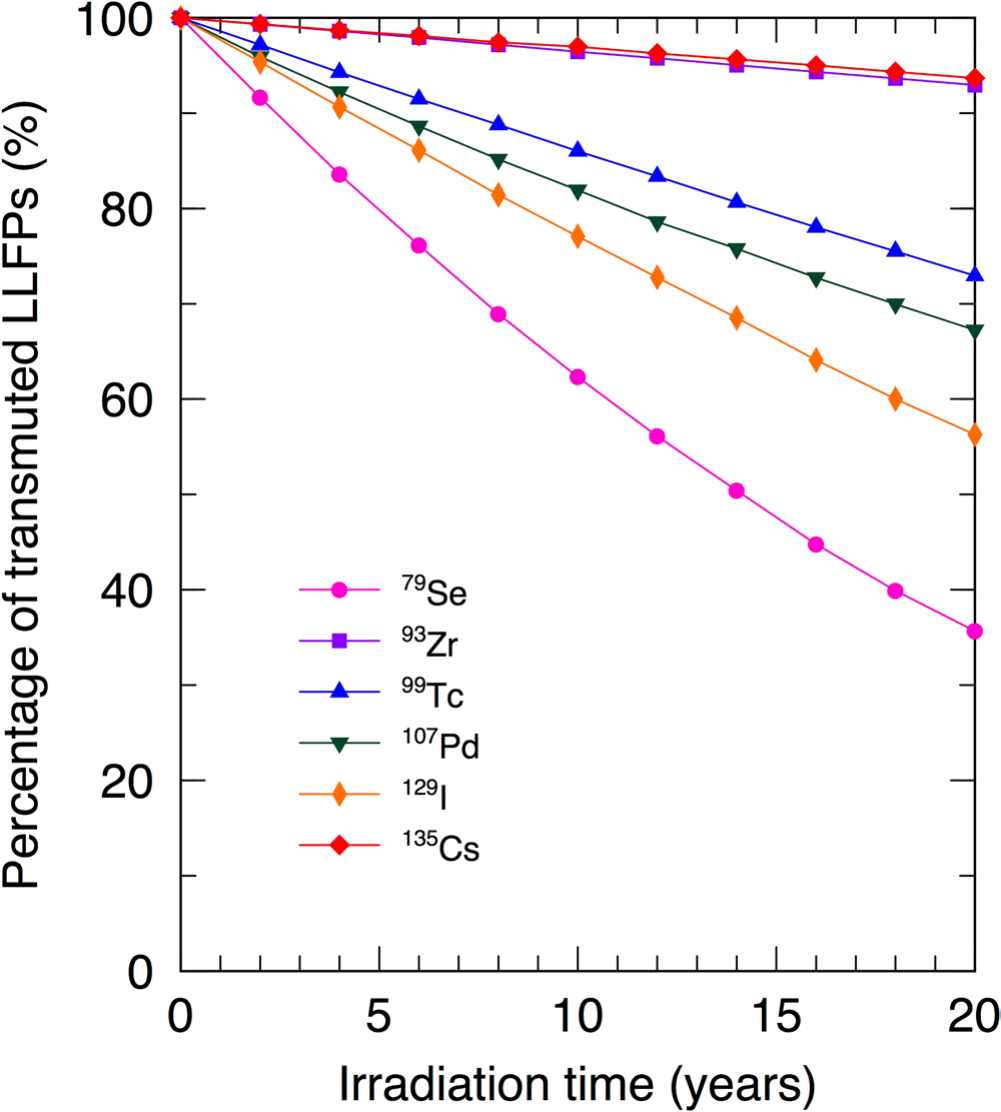
Transmutation of LLFPs over 20 years of irradiation. Irradiation time dependence of the variations of transmuted LLFPs. The percentages of transmuted LLFPs after 20 years are in the order ^135^Cs $\approx$ ^93^Zr < ^99^Tc < ^107^Pd < ^129^I < ^79^Se.

The masses of transmuted LLFPs over 20 years of continuous irradiation were simulated and calculated. Figure 3 shows that all LLFPs decrease approximately linearly with increasing irradiation time. For ^79^Se, ^99^Tc, ^107^Pd, and ^129^I, reductions are more than 25%, whereas reductions are less than 10% over 20 years of irradiation for ^93^Zr and ^135^Cs. This results are due to the considerably smaller neutron capture cross sections of ^93^Zr and ^135^Cs compared to those of other LLFPs. However, the results imply that the neutron capture cross section of averaged thermal and resonance region does not seems to govern the order of transmutation efficiency. The amounts of the other isotopes also vary linearly over 20 years of irradiation (see Supplementary Figures 2–7).

### Transmutation analyses

The approximately linearly decreases of LLFPs (Fig. 3) used to evaluate the effective half-lives, and the TR and SR values for LLFPs were obtained by linear fitting of numerical data of the simulations. Table 2 shows only maximum values and Table 4 in supplementary information shows the maximum, minimum and average rates of transmuted LLFPs. The maximums are calculated from the first irradiation period (0 to 2 years), and the minimums are from the final period (18–20 years). As shown in Table 2, the effective half-lives of the LLFPs decreases substantially to 10^2^ years from natural half-life, which are much shorter than their natural half-lives. The radioactivities of the LLFPs persist with the total radioactivity for over 10^5^ years (see Supplementary Figure 8). The decreases in effective half-lives indicate that the radioactivities of LLFPs will be reduced the time scale to 10^2^ years. These radioactivities will be reduced the time scale to 10^2^ years as the effective half-lives indicated.

**Table 2:** Evaluated parameters obtained from MVP output data of transmutations of LLFPs.

LLFP	Natural half-life [years]	Effective half-life [years]	TR [%/year]	Production [g /years]	Transmutation [g /years]	SR	Loaded	Minimum
^79^Se	3.27 × 10^5^	15.6	3.20	4.20 × 10^1^	4.32 × 10^3^	102.80	54	1
^99^Tc	2.11 × 10^5^	37.0	1.35	5.71 × 10^3^	2.98 × 10^4^	5.21	54	11
^107^Pd	6.5 × 10^6^	0.4	1.65	3.27 × 10^3^	9.27 × 10^3^	2.84	54	20
^129^I	1.57 × 10^7^	22.8	2.19	1.67 × 10^3^	9.05 × 10^3^	5.42	54	10
^93^Zr	1.53 × 10^6^	145.1	0.34	3.60 × 10^3^	1.16 × 10^4^	3.24	498	154
^135^Cs	2.3 × 10^6^	165.2 (357.8)	0.31 (0.14)	1.03 × 10^4^	1.53 × 10^4^ (1.80 × 10^4^)	1.49 (1.75)	498	335 (285)
							Total	531 (481)

(For Cs, number shown in parentheses are the case when the “irradiation and cooling method” is applied).

The TRs seems to be smaller than other proposed systems. The relatively low TRs are acceptable because the key objective of this system is to develop a method to achieve SR > 1.0 for all LLFPs (see Method section for details).

The SRs were calculated from the ratio of the transmuted LLFP mass to the produced LLFP mass in the core fuel. As shown in Table 2, the SRs for ^79^Se, ^99^Tc, ^107^Pd, and ^129^I are sufficiently large (SR > 1) with the conditions that they are loaded in 54 assemblies in the innermost row of the radial blanket region. The SRs for these LLFPs are higher because of their relatively large neutron capture cross sections and small fission yields. In contrast, the SRs for ^93^Zr and ^135^Cs are barely more than 1.0, even though they were loaded in a total 498 assemblies in the radial blanket region (full length) and the radial shield region (core height).

The computational simulations were performed separately for each LLFP, although the simultaneous transmutation of all LLFPs is necessary in practice. The minimum number of assemblies (N_*min*_) required to achieve SR > 1.0 were estimated from the number of loaded assemblies (N_*loaded*_) divided by SR (N_*min*_ = N_*loaded*_/SR) (Table 2). For instance, ^79^Se is loaded in 54 assemblies in the simulation, but the number of assemblies required to achieve SR > 1.0 for the simultaneous transmutation is only 1. For ^93^Zr and ^135^Cs, much more assemblies are required to achieve SR > 1.0 for the simultaneous transmutation of all LLFPs. When the total number of required assemblies is less than the total number of assemblies (498 assemblies), it is possible that SR > 1.0 for the simultaneous transmutation of all LLFPs. Nevertheless, the total number of assemblies required for the simultaneous transmutation exceeded 530. To improve the overall system efficiency, we focus on Cs in the next section because Cs is the most difficult LLFP to transmute.

### Non-continuous transmutation of ^135^Cs

**Figure 4:**
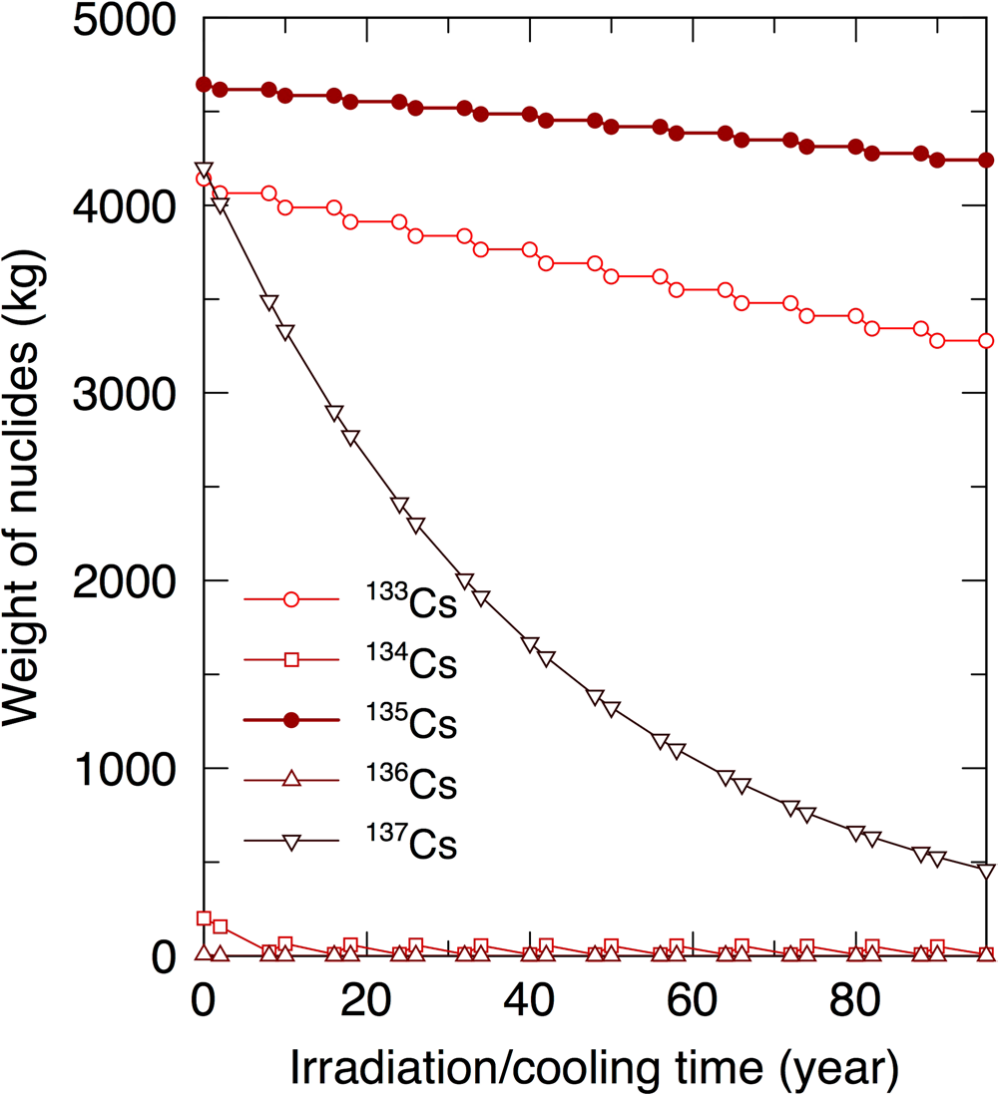
Transmutation of Cs isotopes by the irradiation and cooling method. Irradiation time dependence of the variations of transmuted Cs isotopes using the irradiation and cooling method to prevent the neutron capture of ^134^Cs and additional production of ^135^Cs.

The irradiation and cooling method (see Method section for more details) was used to study a more suitable Cs transmutation scheme. Figure 4 shows the transmutation of Cs isotopes by the “irradiation and cooling method”. The initial compositions of Cs isotopes contain approximately equal amounts of ^133^Cs as ^135^Cs (Table 1). In the reaction chain of Cs isotopes (Supplementary information Figure 9), ^133^Cs produces ^134^Cs by the neutron capture reaction, and then ^134^Cs produces ^135^Cs. Considering the half-life of ^134^Cs (2.0648 years), the irradiation and cooling method, which consists of 2 years of irradiation and 6 years of cooling, was proposed to suppress the production of ^134^Cs, which is a source of ^135^Cs^32^. In this method, the weight of ^135^Cs gradually decreases, meaning that the method would take longer time than continuous irradiation. Nevertheless, when the “irradiation and cooling method” is applied, SRs are >1.0 for all LLFPs and the resulting total number of assemblies required for simultaneous transmutation is comparable with the total number of assemblies considered.

The target nuclides are converted into stable or non-radioactive nuclides during transmutation, and the compositions of nuclides in the target pellets change. Therefore, LLFPs from irradiated pellets under real irradiation must be recycled. In this study, pellet recycling is not considered, although recycling will improve efficiency. Additionally, optimizing the pin and blanket configurations and the LLFP and YD_2_ mixing ratio will also improve the efficiency of the proposed transmutation system.

## Conclusion

We demonstrated that using YD_2_ as a moderator may improve transmutations of LLFPs in the fast spectrum reactor and can be achieved SR > 1.0 for all LLFPs. Neutron spectra in various positions in the reactor were analyzed and the LLFP transmutation capability defined by the effective half-lives, SRs and TRs of LLFPs were evaluated by the Monte Carlo code MVP-II and the burn-up simulation code MVP-BURN with JENDL-4.0 nuclear data library.

YD_2_ was shown to be an effective moderator material for softening the neutron spectrum leaking from the core. This soft spectrum allowed LLFP transmutation in the radial blanket and shield regions. Each LLFP was loaded without isotope separation but with the initial compositions contained all isotopes. The calculations and analyses showed that the half-lives of LLFPs decreased drastically from more than 10^5^ years to less than 10^2^ years. The TRs for LLFPs were relatively small compared to other systems because we focused on improving the SRs. The SRs for all LLFPs were >1.0, including ^135^Cs, which is difficult to transmute effectively. Although the simulations were performed with assuming each LLFP was loaded separately, the proposed system achieved SR > 1.0 for all LLFPs within a total of 481 assemblies when the irradiation and cooling method of Cs transmutation was applied. The number was smaller than the 498 assemblies, which is the maximum number of assemblies by the core specification.

We established an effective, efficient transmutation method for LLFPs to increase transmutation efficiency and improve SRs without isotope separations. If the core region is composed of transuranic or long-life MA nuclides and they can be burned as fuel materials, all the long-lived radioactive elements can be consumed by using the proposed system. In future work, we will examine the simultaneous transmutations of all LLFPs with SR > 1.0. Furthermore, such computational simulation study strongly depends on the nuclear data library regarding nuclear fission and reaction, such as cross section of nuclide, fission product yield, decay chain^47^. We thus also address the improvements of these data by various way of physical descriptions^48,49^.

## Methods

### Computational model and method

The core characteristics was evaluated with a continuous-neutron energy Monte Carlo Code MVP-II/MVP Burn^46^ with JENDL-4.0^45^ cross-section library. The number of history of neutrons was 10,000batch, the number of skipped batches was 100 for accurate source distribution, and the number of effective batches was 1,000. From this Monte Carlo simulation, the neutron spectrum and the reaction rates of LLFPs in various regions in the fast spectrum reactor were obtained. The typical statistical error of the multiplication factor (k_*eff*_) was about 0.015% in 1 $\sigma$ error. The statistical error for reaction rates for all LLFPs at the most inner row of radial blanket are also sufficiently low between 0.1–0.5%, and that at the most outer row of shield region for Cs and Zr are below 1.0%. The burn-up simulations were performed using MVP-BURN code with ChainJ40 burn-up chain data handling actinides from Th to Cm and 193 FPs^46^. The fission product yield data was also derived from JENDL-4.0.

### Reactor core description

The fast reactor core used in this study is a sodium-cooled fast breeder reactor. Figure 1 shows the core layout, assembly configuration, and pin array. Some of the parameters are summarized in Supplementary Table 1. The reactor is simulated to produce a thermal power of about 710 MWt. The core consists of 198 fuel assemblies, 19 control rods, 174 radial blanket assemblies, and 384 radial shielding assemblies in a core height of 930 mm and diameter of 1800 mm. The core fuel assembly contains 169 core fuel pins that are composed of uranium-plutonium MOX fuel pellets covered by stainless steel cladding. The radial blanket is composed of three rows, and the number of assemblies in the innermost (first row), middle (second row), and the outermost (third row) are 54, 60, and 60, respectively. The radial blanket assembly incorporates 61 pins. The primary feature of the radial blanket assembly is to convert fertile fuel (depleted UO_2_) into fissile material by neutron capture reactions. In this system, the radial blanket assemblies are replaced by the assemblies with pins containing the pellets of the mixture of LLFPs and moderator as an alternative for depleted UO_2_ pellets. In the simulations, refueling could not be simulated for the fuel assemblies and LLFPs. Therefore, the core fuel volume was virtually magnified 100-fold to obtain the constant neutron flux from the core for 20 years.

### Selection of LLFPs

In general, the major LLFPs are ^79^Se, ^93^Zr, ^99^Tc, ^107^Pd, ^126^Sn, ^129^I, and ^135^Cs. These nuclides will probably dominate the long-term radioactivity of the geological disposal of SNF (Supplementary Figure 1). Because the total amount of ^126^Sn is small, we have focused on ^79^Se, ^93^Zr, ^99^Tc, ^107^Pd, ^129^I, and ^135^Cs. Transmutation scheme for target LLFPs are as follows:

$$\begin{array}{c}{}^{79}{\rm{S}}{\rm{e}}({\rm{n}},\gamma ){}^{80}{\rm{S}}{\rm{e}}\\ {}^{93}{\rm{Z}}{\rm{r}}({\rm{n}},\gamma ){}^{94}{\rm{Z}}{\rm{R}}\\ {}^{99}{\rm{T}}{\rm{c}}{({\rm{n}},\gamma )}^{100}{\rm{Tc}}\mathop{\longrightarrow }\limits^{{T}_{\mathrm{1/2}}\mathrm{=15.46}\,\,s}\,{}^{100}{\rm{R}}{\rm{u}}\\ {}^{107}{\rm{P}}{\rm{d}}\,{({\rm{n}},\gamma )}^{108}{\rm{Pd}}\\ {}^{129}{\rm{I}}{({\rm{n}},\gamma )}^{130}{\rm{I}}\mathop{\longrightarrow }\limits^{{T}_{1/2}=12.36\,h}\,{}^{130}{\rm{X}}{\rm{e}}\\ {}^{135}{\rm{C}}{\rm{s}}\,{({\rm{n}},\gamma )}^{136}{\rm{Cs}}\mathop{\longrightarrow }\limits^{{T}_{1/2}=13.16\,\,d}\,{}^{136}{\rm{B}}{\rm{a}}\mathrm{.}\end{array}$$

The chemical forms of the LLFPs are an important factor to consider. The material design and fabrication of the irradiation target containing LLFPs and moderator material involve considerations of a wide range of phenomena that affect pellet performance, such as thermal tolerance, swelling, irradiation damage, cracking, radioactive isotope production, and gas evolution. It is difficult to treat LLFPs as the same chemical substance because they have different physical and chemical properties. Assessing a pellet as a function of temperature and irradiation history is a minimum performance standard. The metallic form is the best choice because it generally has high melting point and the space volume for loading can be minimized^50^. Thus, we have chose metallic forms for ^79^Se, ^93^Zr, ^99^Tc and ^107^Pd. The pellets containing these metals were simulated as a homogeneous mixture with the moderator material, YD_2_. For I, our previous study showed that BaI_2_ is the most suitable compound because of its chemical stability and easy manufacturing^50^. For Cs, many Cs compounds have been proposed^51^. In this study, cesium carbonate (Cs_2_CO_3_) was chosen because it has a higher melting point (610 °C) and chemical stability at the ordinary temperature of the fast reactor. We will address the fabrication of LLFP pellets with YD_2_ in a future study.

To transmute LLFPs efficiently and effectively, the LLFPs should be dispersed homogeneously in the pellet^42^. In a previous study, we fabricated Zr-based pellets containing thin Tc metal wires and simulated the homogeneous dispersion of immiscible materials^42^. To simplify and reduce the calculation cost, various ratios with metallic or chemical forms of the target LLFPs were investigated with homogeneous dispersion in YD_2_. The mixing ratios of LLFP/YD_2_ were varied based on the position of the radial blanket or radial shielding assemblies (Supplemental Table 1). These ratios were determined based on our previous studies^52^ although they have not been optimized yet.

The composition of LLFPs obtained from the burn-up simulations of MOX fuel by fast breeder reactor core at 80 GWd/t for 2 years are listed in Table 1. The isotopic compositions in Table 1 were used as the initial composition for LLFPs in the pellet. The transmutations of LLFPs were computationally simulated without isotope separation.

### Assembly configuration design for LLFPs transmutation

The transmutation capability strongly depends on the neutron capture cross sections of the LLFPs. The neutron capture cross sections in the electronvolt to kiloelectronvolt region of LLFPs (Supplementary Figure 2) allow the transmutation of the LLFPs by softened neutron spectra in the blanket and shield regions. The amounts of LLFPs that need to be transmuted are different for each element. Two types of arrangement of the radial blanket assembly were used depending on their transmutation capability. The overall length of the assembly consists of the fuel length (core height), the upper and lower blankets, the fission gas plenum, and other structural objects. The full length includes the total length of the fuel length and the upper and lower blankets. For Se, Tc, Pd, and I (Type-1), LLFPs were loaded in 54 of the radial blanket assemblies in the innermost row (core height). For Zr and Cs (Type-2), LLFPs were loaded in 498 assemblies in the radial blanket (full length) and shield region (core level). The total irradiation period was determined by the life-time of the LLFP assemblies, which was evaluated as 20 years. However, 20 years of irradiation is an unrealistic scenario for a real nuclear reactor owing to the irradiation damage to the constituent materials. However, our simulation is the worst-case scenario for the pellets containing LLFPs. The isotope composition in the pellets changes during irradiation. The pellets will be recycled so that the other elements produced by transmutation are removed.

### Transmutation of cesium

Cesium has the stable isotope ^133^Cs, short-lived ^134^Cs and ^136^Cs, and long-lived ^135^Cs and ^137^Cs (Supplementary Figure 9). ^133^Cs produces ^134^Cs by the neutron capture reaction, and then ^134^Cs produces ^135^Cs in the same way. Consequently, at the beginning of the irradiation, the loaded ^133^Cs produces ^134^Cs, which becomes the source of ^135^Cs. We developed the irradiation and cooling method based on this sequence^31^. After 2 years of irradiation, ^133^Cs is transmuted into ^134^Cs, which decays with a half-life of 2.0648 years. A cooling interval 6 years after irradiation allows ^134^Cs to decay into stable ^134^Ba. After ^134^Cs decays out, irradiation of ^135^Cs is followed by decay into stable ^136^Ba. Cs transmutation is achieved by repeating 2 years of irradiation and 6 years of cooling. In this study, the production and neutron capture of ^134^Ba, ^136^Ba, and ^137^Ba (produced by the decay of ^137^Cs) were neglected because they decrease the SRs slightly.

### Effective half-life, transmutation ratio and support ratio

The effective half-life is an important parameter for evaluating the efficiency of transmutation. The effective half-life is the half-life when transmutation is considered. For a single target nuclide, the effective half-life can be defined as

1 $$Effective\,half\,life\equiv \frac{ln2}{\lambda +\sigma \phi },$$

where $\lambda$, $\sigma$, and $\phi$ are the decay constant of the target LLFP, the effective neutron capture cross section, and the neutron flux, respectively. If the $\lambda$ values of LLFPs are small, linear expression can be applied. In this system,

2 $$Effective\,half\,life\approx \frac{ln2}{\sigma \phi }\mathrm{.}$$

Other two important parameters of the transmutation system are Transmutation Rate (TR) and Support Ratio (SR). TR is defined as the ratio of the amount of initial loaded LLFPs to the amount of transmuted LLFPs in the target assembly (per unit time) as

3 $$Transmutation\,rate(TR)\equiv \frac{N\mathrm{(0)}-N(T)}{TN\mathrm{(0)}},$$

where N(0) and T are the number of initial atoms of a LLFP in the target assemblies and the irradiation period, respectively. If the burn-up chain of a LLFP only has a capture reaction,

4 $$TR \sim \frac{N\mathrm{(0)}-N(T)exp(-\sigma \phi T)}{TN\mathrm{(0)}}=\frac{1-exp(-\sigma \phi T)}{T} \sim \frac{1-\mathrm{(1}-\sigma \phi T+\cdot \cdot \cdot )}{T}$$

and if the value of $\sigma \phi$ T is small enough,

5 $$TR\approx \sigma \phi .$$

In this system, TR of LLFPs except for ^135^Cs can be approximately expressed by Eq. 5.

SR is defined as the ratio of the amount of transmuted LLFPs to the amount of LLFPs produced in the core fuel over the same period of time in a reactor,

6 $$Support\,ratio(SR)\equiv \frac{N\mathrm{(0)}-N(T)}{\gamma MT},$$

where $\gamma$ and M are the LLFP yield per fission of fuel materials and the total fission rate of the core, respectively. If the burn-up chain of a LLFP only has a capture reaction,

7 $$SR \sim \frac{N\mathrm{(0)}-N\mathrm{(0)}exp(-\sigma \phi T)}{\gamma MT}=\frac{N\mathrm{(0)}\{1-exp(-\sigma \phi T)\}}{\gamma MT} \sim \frac{N\mathrm{(0)}\{1-\mathrm{(1}-\sigma \phi T+\cdot \cdot \cdot )\}}{\gamma MT}$$

and if the value of $\sigma \phi$ T is small enough,

8 $$SR\approx \frac{N\mathrm{(0)}\sigma \phi }{\gamma M}\mathrm{.}$$

In this system, SR except for ^135^Cs can be approximated by Eq. 8. The easiest way to increase SR is to increase the amount of initially loaded LLFPs. However, when N(0) is increased, $\sigma \phi$ is decreased by the neutron self-shielding effect in the target. The diluted LLFP targets increase the TRs due to smaller neutron flux depression, thus the two effects oppose each other. This is why the fast spectrum reactor systems in many previous studies could not transmute LLFPs simultaneously with SR > 1.0. If SR > 1.0 can be achieved, the self-produced LLFPs could be transmuted during electric power generation.

## Electronic supplementary material

Supplemental information
