# Publisher Correction: Method to Reduce Long-lived Fission Products by Nuclear Transmutations with Fast Spectrum Reactors

**DOI:** 10.1038/s41598-017-17100-y

**Published:** 2017-12-05

**Authors:** Satoshi Chiba, Toshio Wakabayashi, Yoshiaki Tachi, Naoyuki Takaki, Atsunori Terashima, Shin Okumura, Tadashi Yoshida

**Affiliations:** 1 Laboratory for Advanced Nuclear Energy, Tokyo Institute of Technology, 2–12–1 Ookayama, Meguro-ku, Tokyo, 152–8550 Japan; 2 Tohoku University, 2–1–1 Katahira, Aoba-ku, Sendai, Miyagi, 980–8577 Miyagi, Japan; 3 Oarai Research and Development Center, Japan Atomic Energy Agency, 4002, Narita-cho, Oaraimachi, Ibaraki, 311–1393 Japan; 4 Department of Nuclear Safety Engineering, Tokyo City University, 1–28–1 Tamazutsumi, Setagaya-ku, Tokyo, 158–8557 Japan

*Scientific Reports* **7**:13961; doi:10.1038/s41598-017-14319-7; Article published online 24 October 2017

The original version of this Article contained typographical errors in the Abstract.

“With the YD_2_ moderator in the radial blanket and shield regions, effective half-lives drastically decreased from 106 to 102 years and the support ratios reached 1.0 for all six LLFPs”.

now reads:

“With the YD_2_ moderator in the radial blanket and shield regions, effective half-lives drastically decreased from 10^6^ to 10^2^ years and the support ratios reached 1.0 for all six LLFPs”.

This has now been corrected in the PDF and HTML versions of the Article.

