# Supplementary material for: Method to Reduce Long-lived Fission Products by Nuclear Transmutations with Fast Spectrum Reactors
Source: Sci Rep. 2017 Oct 24;7:13961. doi: 10.1038/s41598-017-14319-7 (PMC5654822; doi:10.1038/s41598-017-14319-7)
Supplement: Supplementary file 1 — Supplemental information [file 41598_2017_14319_MOESM1_ESM.pdf]

Supplementary information

Method to Reduce Long-lived Fission Products by Nuclear Transmutations with Fast Spectrum Reactors

Satoshi CHIBA[1,*], Toshio WAKABAYASHI[2], Yoshiaki TACHI[3], Naoyuki TAKAKI[4], Atsunori TERASHIMA[1], Shin OKUMURA[1], and Tadashi YOSHIDA[1]

[1]Laboratory for Advanced Nuclear Energy, Tokyo Institute of Technology, 2-12-1 Ookayama, Meguro-ku, Tokyo 152-8550, Japan.

[2]Tohoku University, 2-1-1 Katahira, Aoba-ku, Sendai, Miyagi 980-8577, Miyagi, Japan

[3]Oarai Research and Development Center, Japan Atomic Energy Agency, 4002, Narita-cho, Oaraimachi, Ibaraki 311-1393, Japan.

[4]Department of Nuclear Safety Engineering, Tokyo City University, 1-28-1 Tamazutsumi, Setagaya-ku, Tokyo 158-8557, Japan.

[*] chiba.satoshi@nr.titech.ac.jp

Table 1 Key technical parameters of the core used in the simulation

Main parameters	Data used in this study
Thermal power (MW_t)	710
Core height (mm)	930
Number of core fuel assemblies	108, 90, 198 (inner, outer, total)
Number of pins in core fuel assemblies	169
Core pin diameter (mm)	6.5
Core pellet diameter (mm)	5.8
Pu enrichment (%)	22, 30 (inner, outer)
Number of control rods	19
Number of radial blanket assemblies	174
Number of pins in the radial blanket assembly	61
Radial pin diameter (mm)	11.7
Radial pellet diameter (mm)	10.4
Number of shielding assemblies	324
Number of assemblies for Se, Tc, Pd, and I (Type 1)	54
Number of assemblies for Cs and Zr (Type 2)	498
LLFP/$YD_{2.0}$ volume ratio (1st row, for Se, Tc, Pd, I, Zr, and Cs)	70/30
LLFP/$YD_{2.0}$ volume ratio (2nd row, for Zr and Cs)	80/20
LLFP/$YD_{2.0}$ volume ratio (3rd row, for Zr and Cs)	90/10
LLFP/$YD_{2.0}$ volume ratio (shielding assembly for Zr and Cs)	100/0

Table 2 Neutron capture cross sections at 0.0255 eV and resonance integrals[1]

	^{79}Se	^{93}Zr	^{99}Tc	^{107}Pd	^{129}I	^{135}Cs
Neutron capture cross section at 0.0253 eV (barn)	50.02	2.239	23.60	9.243	30.32	8.302
Neutron capture cross section of resonance integral (barn)	108.7	18.20	323.8	112.9	33.25	53.52

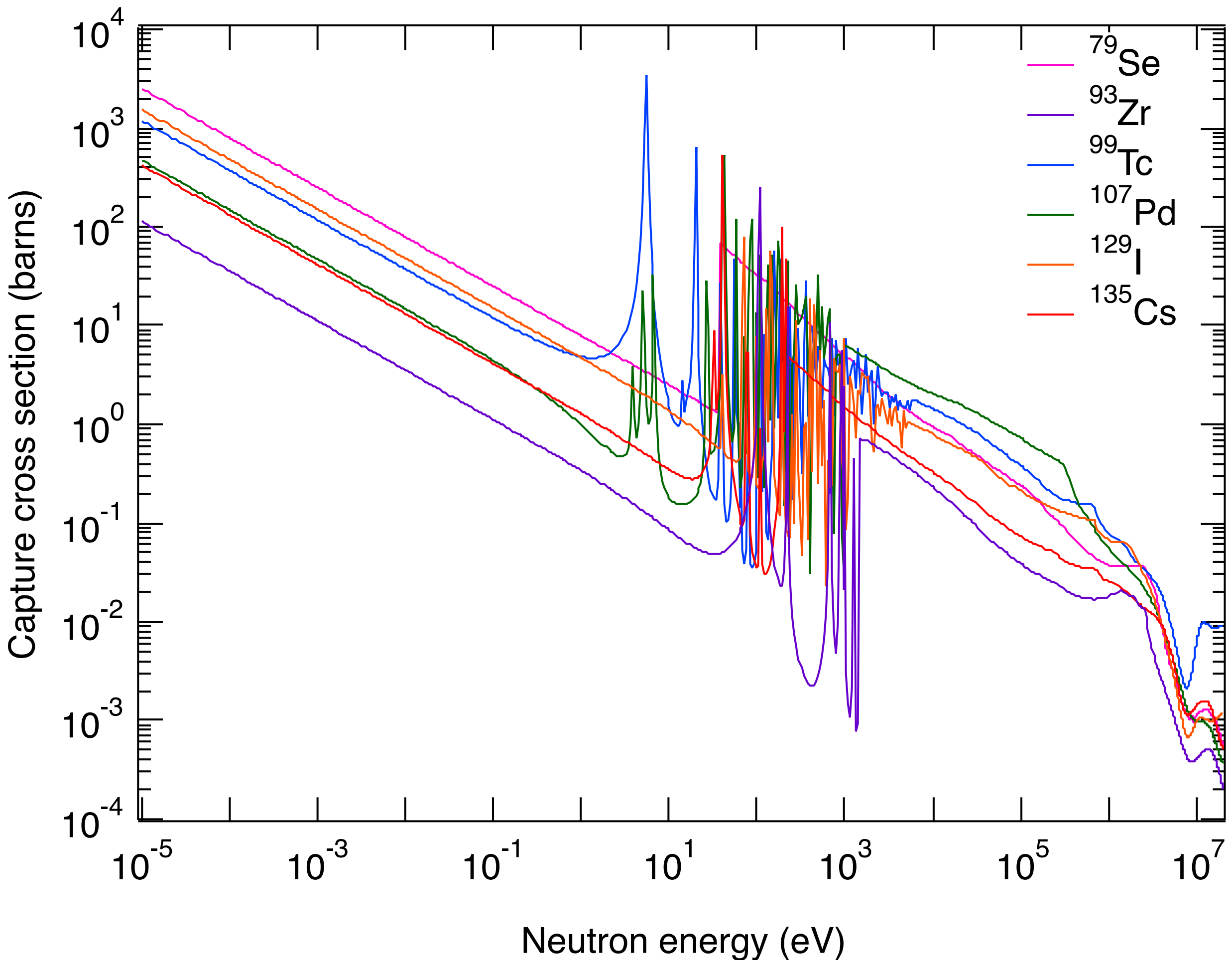

Figure 1 Neutron energy dependency of capture cross sections of LLFPs. | Neutron capture reaction cross sections are important for studying nuclear transmutation of LLFPs. The LLFP neutron cross sections are limited and need their accuracy improved. The data plots are from JENDL-4.0u[1].

Table 3 Integrals of the microscopic capture reaction rate with and without the YD_2 moderator

	^{79}Se	^{93}Zr	^{99}Tc	^{107}Pd	^{129}I	^{135}Cs
With YD_2	1.22×10^{-8}	2.52×10^{-9}	8.96×10^{-9}	1.48×10^{-8}	5.09×10^{-9}	4.24×10^{-9}
Without YD_2	5.86×10^{-9}	1.13×10^{-9}	5.31×10^{-9}	8.90×10^{-9}	3.18×10^{-9}	2.01×10^{-9}
Ratio	2.16	2.23	1.69	1.67	1.60	2.11

Table 4 Evaluated parameters obtained from MVP output data for the LLFP transmutation calculations.

	Natural half-life [years] [1]	Effective half-life [years] [2]	TR[3] [%/year]	Production [g/years][4]	Transmutation [g/years][5]	SR[6]	Loaded[7]	Minimum[8]
					Max. 4.32×10^{3}[(A)]	102.80		1
^{79}Se	3.27×10^{5}	15.6	3.20	4.20×10^{1}	Ave. 3.35×10^{3}	79.72	54	1
					Min. 2.39×10^{3}[(C)]	56.95		1
					Max. 2.98×10^{4}[(A)]	5.21		11
^{99}Tc	2.11×10^{5}	37.0	1.35	5.71×10^{3}	Ave. 2.82×10^{4}	4.94	54	11
					Min. 2.64×10^{4}[(C)]	4.63		12
					Max. 9.27×10^{3}[(A)]	2.84		20
^{107}Pd	6.5×10^{6}	30.4	1.65	3.27×10^{3}	Ave. 7.59×10^{3}	2.32	54	24
					Min. 5.97×10^{3}[(C)]	1.83		30
					Max. 9.05×10^{3}[(A)]	5.42		10
^{129}I	1.57×10^{7}	22.8	2.19	1.67×10^{3}	Ave. 8.26×10^{3}	4.95	54	11
					Min. 7.33×10^{3}[(C)]	4.39		13
					Max. 1.16×10^{4}[(B)]	3.24		154
^{93}Zr	1.53×10^{6}	145.1	0.34	3.60×10^{3}	Ave. 1.14×10^{4}	3.17	498	158
					Min. 1.11×10^{4}[(A)]	3.07		162
					Max. 1.53×10^{4}[(A)]	1.49		335
					(1.80×10^{4}[(D)])[9]	(1.75)[9]		(285)[9]
^{135}Cs	2.3×10^{6}	165.2	0.31	1.03×10^{4}	Ave. 1.45×10^{4}	1.41	498	354
		(357.8)[9]	(0.14)[9]		(1.67×10^{4})[9]	(1.62)[9]		(308)[9]
					Min. 1.40×10^{4}[(C)]	1.36		367
					(1.40×10^{4}[(A)])[9]	(1.36)[9]		(367)[9]
							Max.	531
								(481)[9]
						Total	Ave.	559
								(513)[9]
							Min.	585
								(585)[9]

1) Natural half-life of the target LLFPs.

2) Effective half-life is defined as the time required for the residual amount of LLFP to decrease to half of the loaded LLFP during the burn-up.

3) TR is defined in equation (5). Irradiation period is 20 years for ^{79}Se, ^{93}Zr, ^{99}Tc, ^{107}Pd, and ^{129}I, and 24 years (12th cycle of irradiation and cooling) for ^{135}Cs.

4) Production weight of LLFPs in the core fuel during transmutation.

5) Transmutation weight of LLFPs in the radial blanket assemblies obtained by the calculation from irradiation periods of (A) 0 to 2 years, (B) 2 to 4 years, (C) 18 to 20 years, and (D) 72 to 74 years.

6) SR is defined in equation (8).

7) Number of assemblies loaded with LLFPs in the radial blanket region (and radial shielding region for Zr and Cs).

8) Minimum number of radial blanket assemblies when SR = 1.0 is assumed.

9) When the irradiation and cooling method is applied.

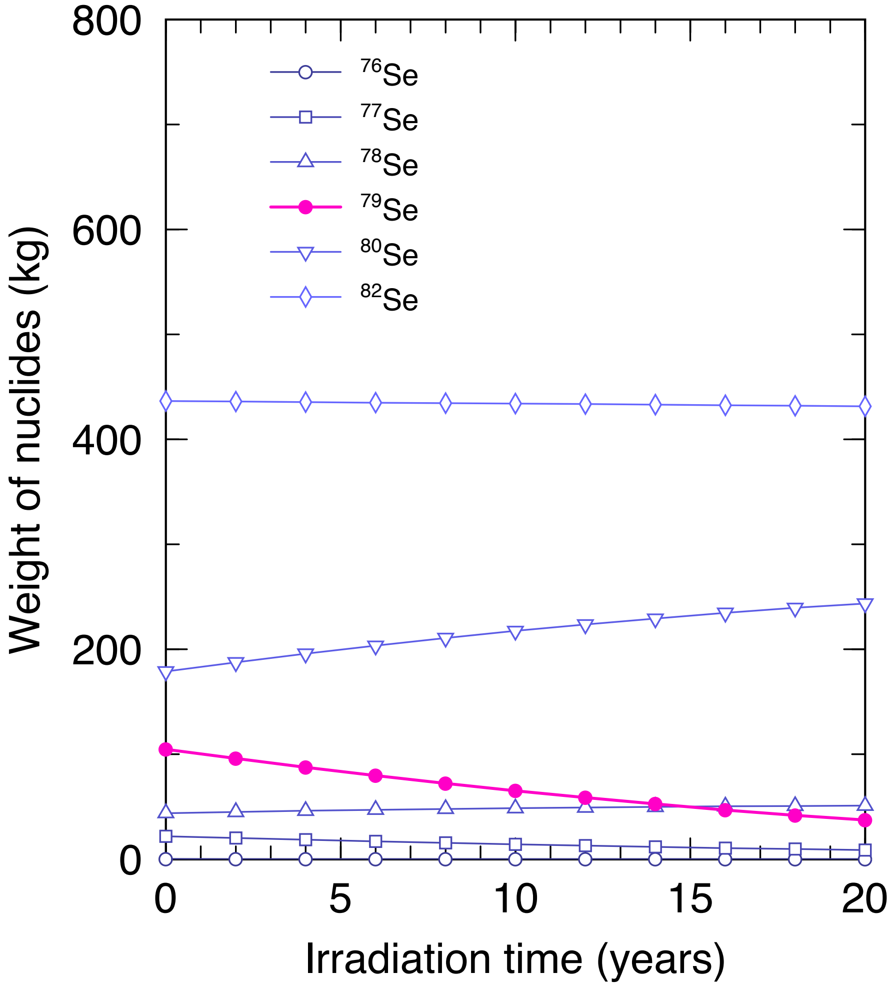

Figure 2 Changes in the weight of Se isotopes over 20 years of irradiation. Irradiation time dependence of the variations of transmuted Se isotopes. One of the LLFPs, ^{79}Se, is effectively reduced after 20 years of irradiation with proposed method.

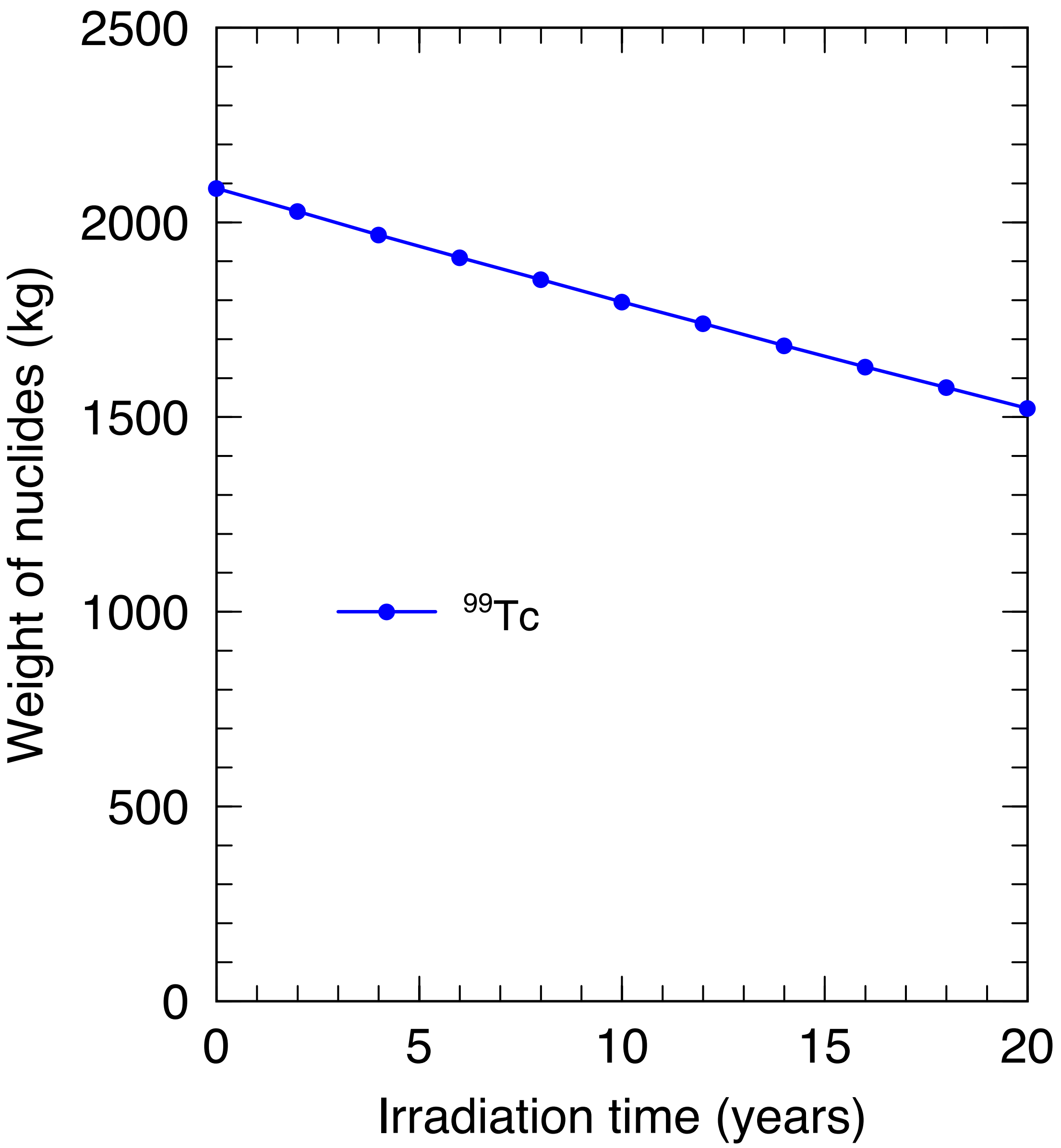

Figure 3 Changes in the weight of Tc over 20 years of irradiation. Irradiation time dependence of the variations of transmuted ^{99}Tc. For the Tc isotopes, only ^{99}Tc, which is one of the LLFPs, is produced in the reactor core. ^{99}Tc can be reduced effectively by 20 years of irradiation with proposed method.

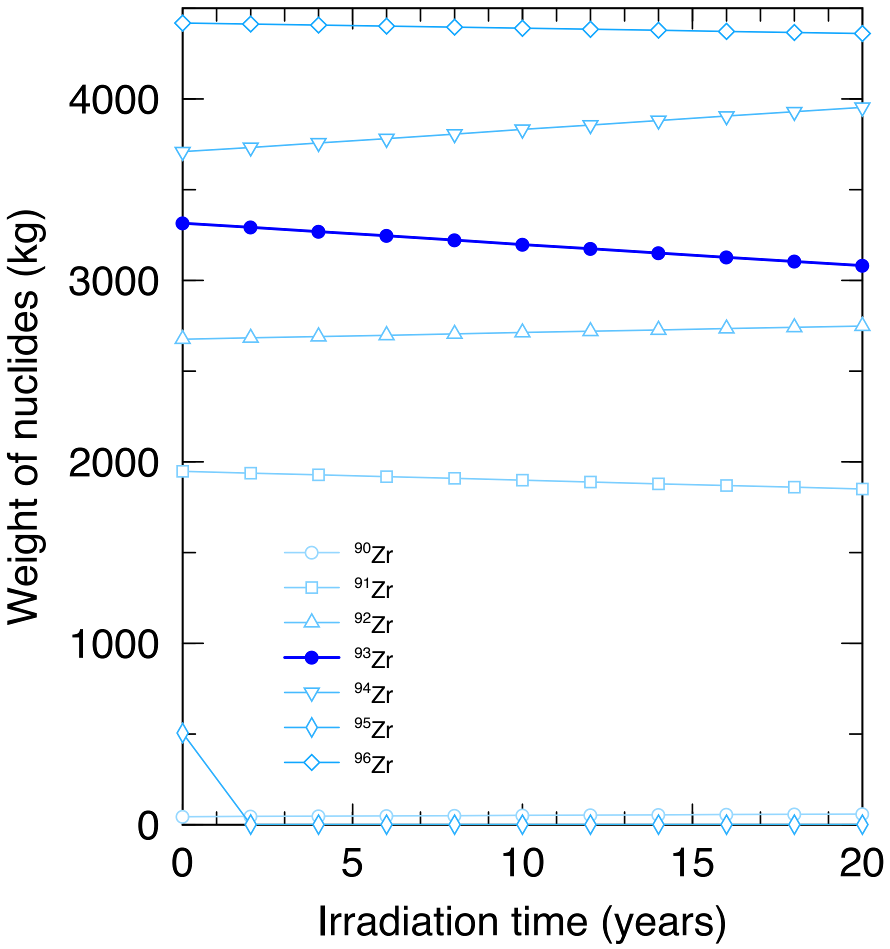

Figure 4 Changes in the weight of Zr isotopes over 20 years of irradiation. Irradiation time dependence of the variations of transmuted Zr isotopes. ^{93}Zr is one of the LLFPs, can be reduced by 20 years of irradiation with proposed method slightly but with achieving SR>1.

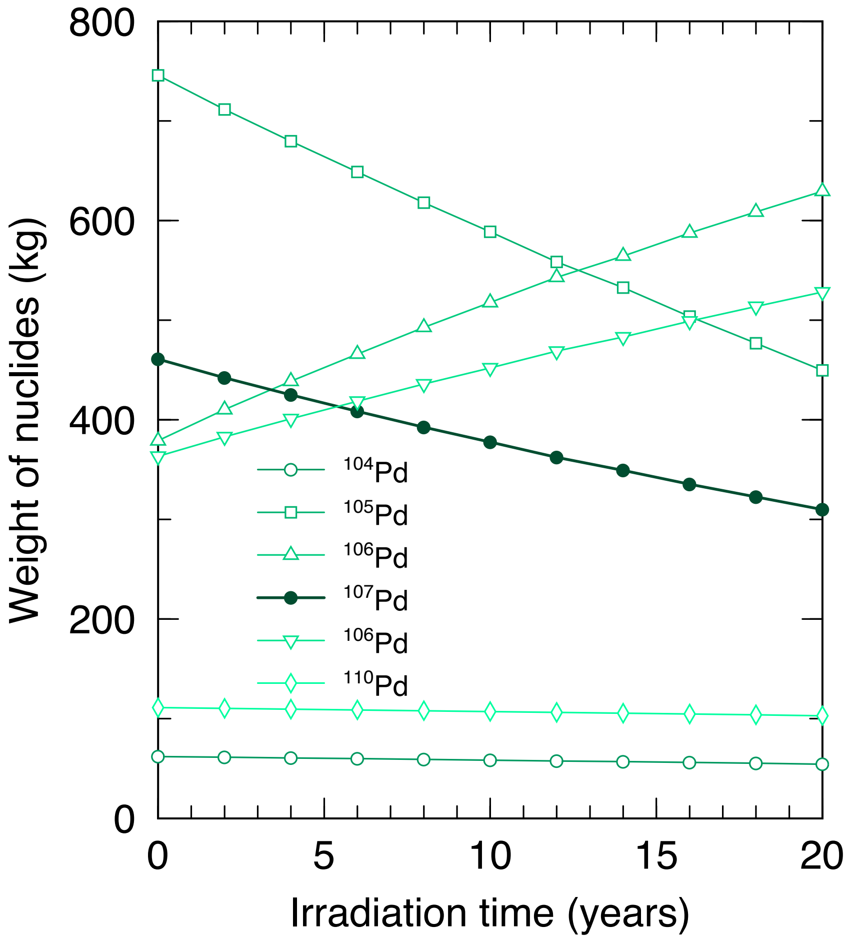

Figure 5 Changes in the weight of Pd isotopes over 20 years of irradiation. Irradiation time dependence of the variations of transmuted Pd isotopes. One of the LLFPs, ^{107}Pd, is effectively reduced after 20 years of irradiation with proposed method.

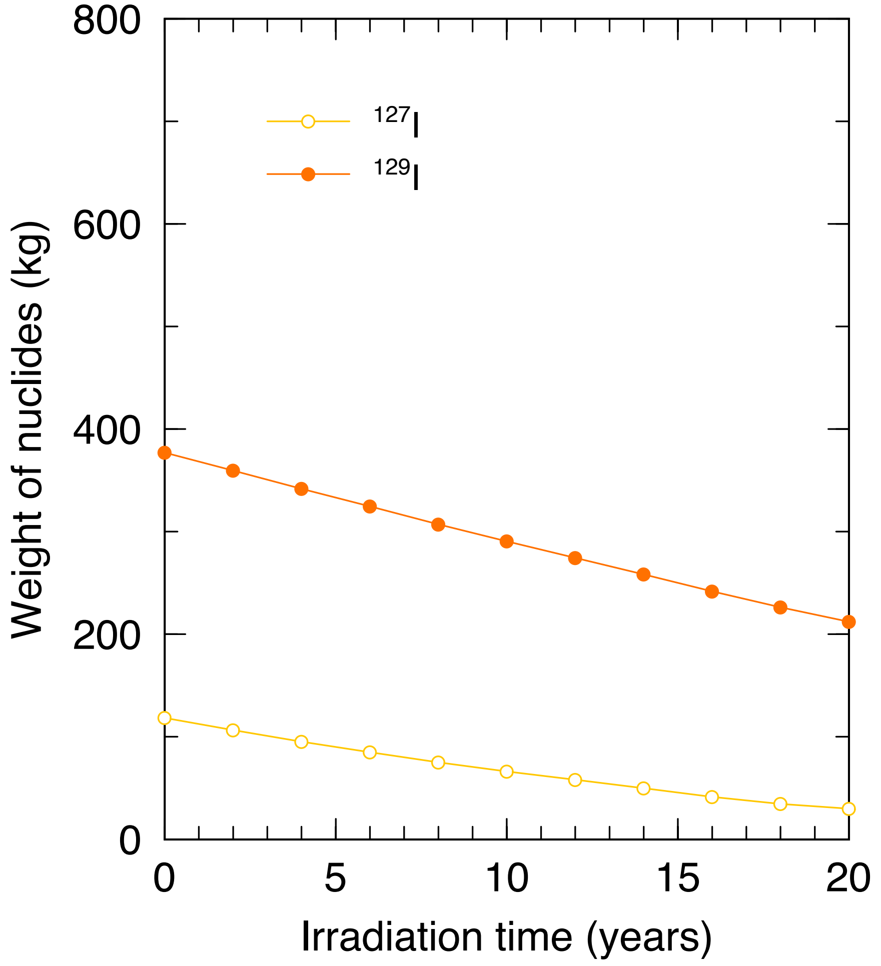

Figure 6 Changes in the weight of I isotopes over 20 years of irradiation. Irradiation time dependence of the variations of transmuted I isotopes. One of the LLFPs, ^{129}I, is effectively reduced after 20 years of irradiation with proposed method.

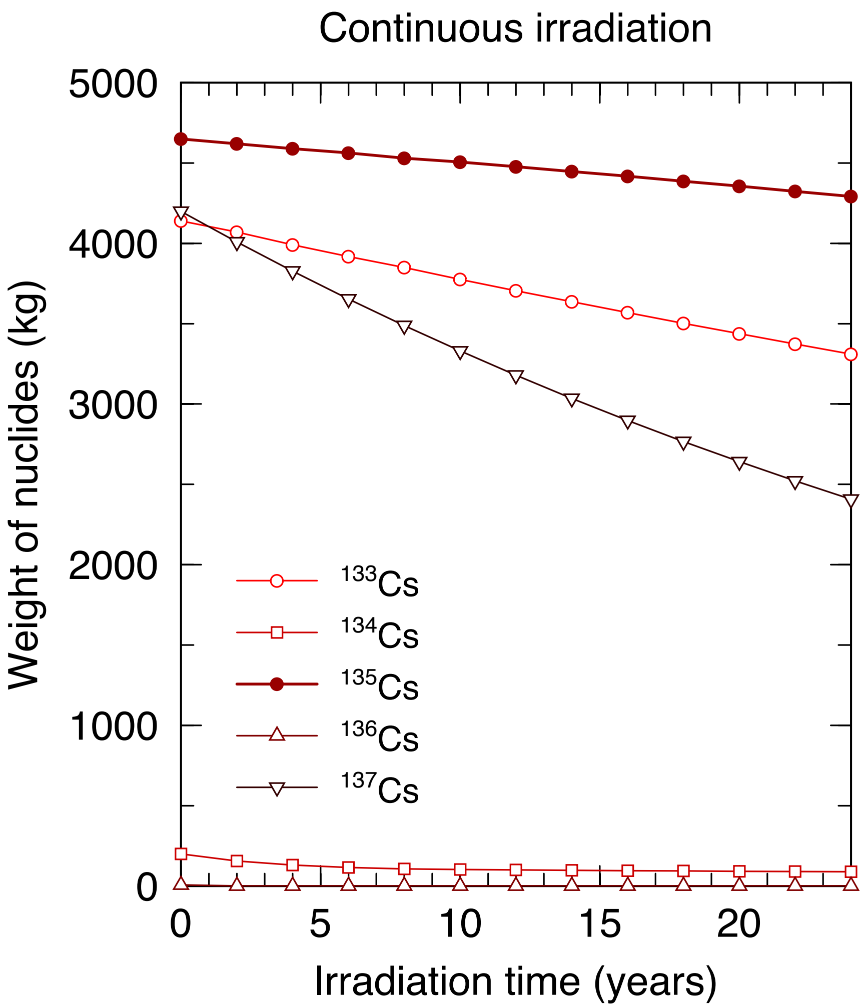

Figure 7 Changes in the weight of Cs isotopes over 20 years of irradiation. Irradiation time dependence of the variations of transmuted Cs isotopes. ^{137}Cs, one of the LLFPs, can be slightly reduced by 20 years of irradiation with proposed method.

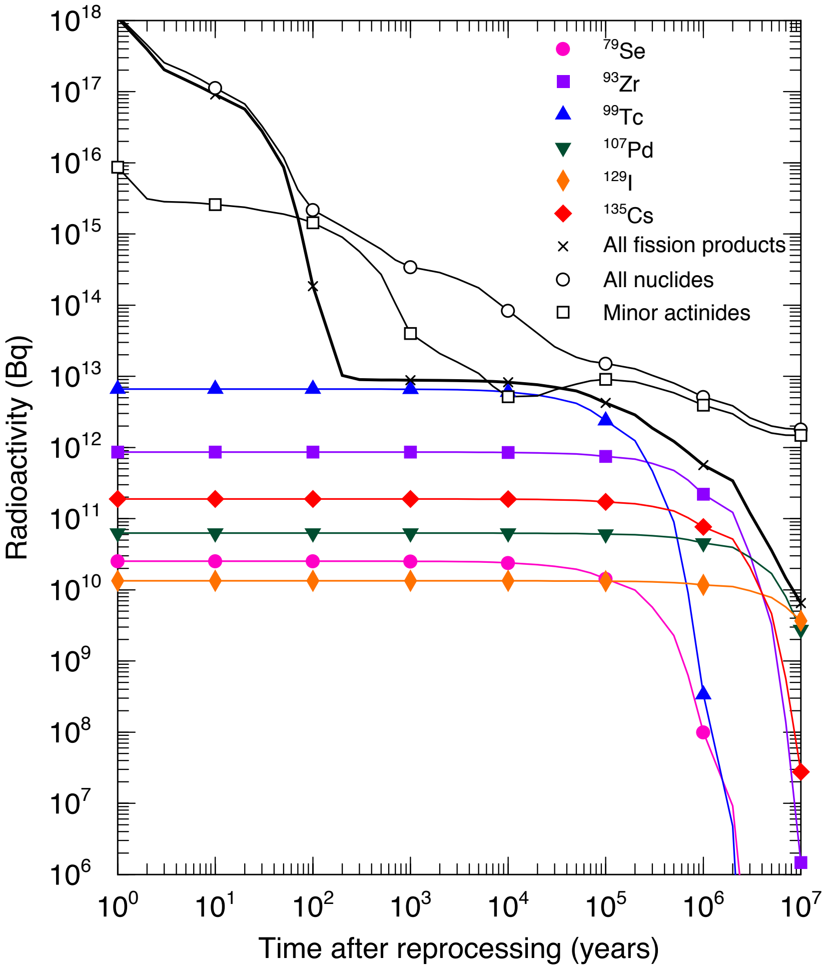

Figure 8 Radioactivity of long-lived fission products (LLFPs) and minor actinides after reprocessing of 1 ton of discharged nuclear fuel calculated by ORIGEN2[2]. | The radioactivity of all fission products dominates the total radioactivity during the first 100 years. Long-term radiotoxicity is dominated by minor actinides and LLFPs.

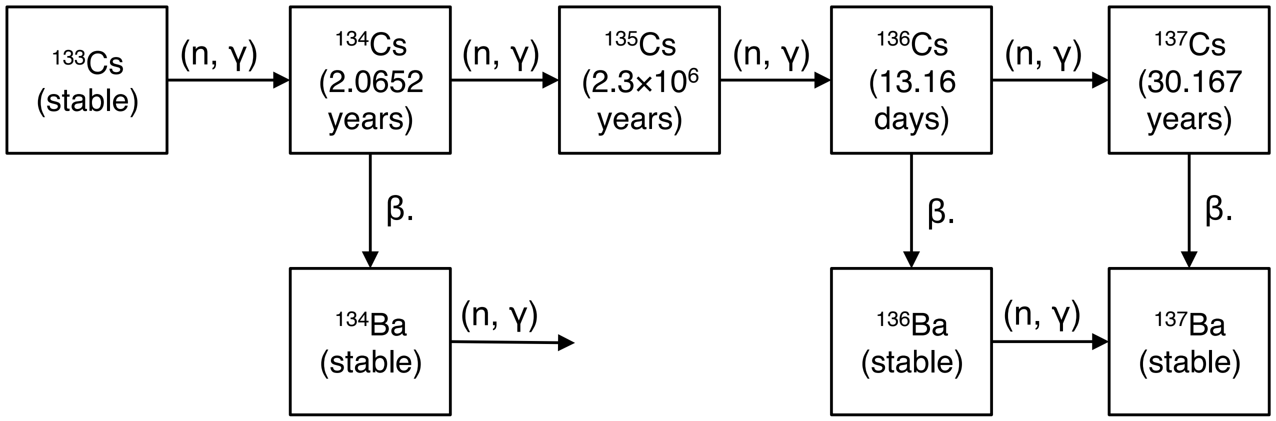

Figure 9 Reaction chain of Cs isotopes.

[1] K. Shibata, *et al*. JENDL-4.0: A new library for nuclear science and engineering. *J. Nucl. Sci. Technol.* **48**, 1–30 (2011).
[2] A. G. Croff, "ORIGEN2: A Versatile Computer Code for Calculating the Nuclide Compositions and Characteristics of Nuclear Materials", *Nucl. Technol.* **62**, 335-352 (1983).
